# Older urban rats are infected with the zoonotic nematode *Angiostrongylus cantonensis*

**DOI:** 10.1016/j.crpvbd.2024.100179

**Published:** 2024-05-21

**Authors:** Phoebe Rivory, Miguel Bedoya-Pérez, Michael P. Ward, Jan Šlapeta

**Affiliations:** a Sydney School of Veterinary Science, Faculty of Science, The University of Sydney, New South Wales, 2006, Australia; b The Lambert Initiative for Cannabinoid Therapeutics, The University of Sydney, Sydney, New South Wales, Australia; c School of Psychology, Faculty of Science, The University of Sydney, Sydney, New South Wales, Australia; d Sydney Infectious Diseases Institute, The University of Sydney, Sydney, New South Wales, Australia

**Keywords:** Angiostrongyliasis, *Angiostrongylus cantonensis*, *cox*1, *Rattus*, Sydney

## Abstract

Rats, being synanthropic, are hosts to agents of zoonotic diseases that pose a threat to human and domestic animal health. The nematode parasite *Angiostrongylus cantonensis*, commonly known as the rat lungworm, is no exception; it can cause potentially fatal neural disease in humans, dogs and other species. The distribution of *A. cantonensis* (haplotypes SYD.1 and Ac13) and its close relative, *Angiostrongylus mackerrasae* is not well understood in Australia. We investigated the prevalence of *Angiostrongylus* in rats in Sydney, Australia, primarily *via* faecal qPCR, and identified the species and haplotypes using partial *cox*1 sequencing. We found a moderate prevalence of infection (29%; 95% CI: 16.1–46.6%) in black (*Rattus rattus*) and brown (*Rattus norvegicus*) rats around public parks and residential areas. This study demonstrates that Sydney’s urban rat population is a reservoir for *A. cantonensis*. Modelling infection status as a function of rat species, sex, tibia length (as a proxy for age), and health index (a measure of weight by size) revealed that older rats are statistically more likely to be infected (*χ*^2^_1_ = 5.331, *P* = 0.021). We observed a dominant presence of the *A. cantonensis* SYD.1 haplotype, for which the implications are not yet known. No *A. mackerassae* was detected, leading us to suspect it may have a more restricted host- and geographical range. Overall, this study illustrates the presence and potential risk of *A. cantonensis* infection in Sydney. Public education regarding transmission routes and preventative measures is crucial to safeguard human and animal health.

## Highlights

• *Angiostrongylus cantonensis* found in 29% of urban rats in Sydney, Australia.
• Infected rats were older, and trapped around parks, gardens and residential areas.
• Only the *A. cantonensis* SYD.1 *cox*1 haplotype was detected.

## Graphical abstract

**Figure:**
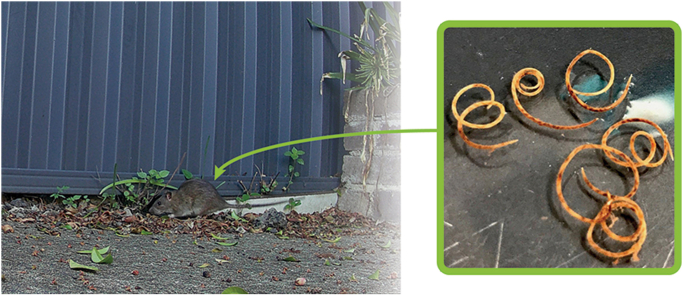

## 1 Introduction

Urban environments represent complex ecosystems. The harbour city of Sydney, New South Wales (NSW) Australia, with over 5 million inhabitants, is a prime example; this densely populated city is estimated to share its urban landscape with approximately 1 million domestic dogs and at least 500 million rats. Synanthropic rats in Australia, namely the invasive black (*Rattus rattus*) and brown (*Rattus norvegicus*) rats, have successfully integrated themselves closely to humans. Rats share more zoonotic pathogens with humans than other animal taxa (Gibb et al., 2020) and act as reservoirs for a vast variety of pathogens, including viruses, bacteria, protozoans and helminths (Himsworth et al., 2013).

One helminth parasite of concern is the rat lungworm, *Angiostrongylus cantonensis* (Chen, 1935). This zoonotic nematode is the causative agent of neural angiostrongyliasis, a serious parasitic disease affecting the central nervous system (CNS) due to neural *larva migrans* (Wang et al., 2008; Barratt et al., 2016). Consequently, *A. cantonensis* is the leading cause of human eosinophilic meningitis globally (Wang et al., 2012). The parasite’s life-cycle involves definitive (rat) and intermediate (mollusc) hosts. Rats and accidental hosts, such as humans and dogs, become infected by ingesting the larvae that develop within infected molluscs (Cowie, 2013). While human infections with *A. cantonensis* are relatively infrequent in Australia (Berkhout et al., 2019; Prociv and Carlisle, 2001), canine neural angiostrongyliasis is recognised as an emerging disease among domestic dogs throughout Australia’s east coast, including in Sydney (Mason, 1987; Lunn et al., 2012; Lee et al., 2021). Notably, recent studies revealed the existence of two haplotypes (Ac13 and SYD.1) in several clinical dog cases (Mallaiyaraj Mahalingam et al., 2021; Baláž et al., 2023). Ac13 and SYD.1 are grouped within a clade, including other haplotypes that have invaded new regions (Červená et al., 2019; Tian et al., 2023). Whilst investigations into the causative haplotype in canine cases continue, the Ac13 haplotype has been detected marginally more often (10/15; Mallaiyaraj Mahalingam et al., 2021; Baláž et al., 2023).

Following an investigation into a fatal case of neural angiostrongyliasis in a captive primate in western Sydney, Rivory et al. (2023) reported a concerningly high prevalence of the *A. cantonensis* Ac13 haplotype in brown rats (*R. norvegicus*). Indeed, it was the Ac13 haplotype that was confirmed in the primate post-mortem (Rivory et al., 2023). The interaction between the haplotypes and their permissiveness in various definitive and intermediate hosts, and therefore the risk for accidental infection, is not understood. The dominance of Ac13 in western Sydney’s rat population and eastern Australia’s canine angiostrongyliasis cases suggests there may be phenotypic differences between the two haplotypes.

Australia has a native rat lungworm species, *Angiostrongylus mackerrasae*, which shares a similar life-cycle and larval neurotropism with *A. cantonensis* (Bhaibulaya, 1968, 1975). However, despite a single documented case in a flying fox (Mackie et al., 2013), definitive identification of *A. mackerrasae* in accidental host angiostrongyliasis cases remains elusive. Despite more intensive efforts to speciate *Angiostrongylus* using ITS-2 deep-sequencing (Baláž et al., 2023), the apparent absence of *A. mackerrasae* in clinical cases may be attributed to insufficient efforts to determine the causative species, or a genuine lack of pathogenicity in accidental hosts (Prociv et al., 2000). Regardless, *A. mackerrasae* is still considered a potential agent of neural angiostrongyliasis (Prociv et al., 2000).

This study utilises faecal qPCR to determine if Sydney’s urban rats act as hosts for *Angiostrongylus* and, if so, to model infection status using rat species, sex, tibia length (as a proxy for age) and health index (a function of the rat’s weight according to its size) as predictors. We aim to confirm the *Angiostrongylus* spp. and haplotypes present *via* partial *cox*1 sequencing.

## 2 Materials and methods

### 2.1 Rat sample collection

Black (*R. rattus*) and brown (*R. norvegicus*) rats were trapped during the years of 2020 and 2021 in the City of Sydney local government area (LGA) for a leptospirosis surveillance project (University of Sydney Animal Ethics Committee approval number 2020/1725, N00/1–2013/3/5920, 2017/1130 and 2019/1665). The methodology for rat trapping, placement of traps and morphometric data collection are described in the materials and methods of the original article by Bedoya-Pérez et al. (2023). Superfluous faecal samples from the abovementioned project were provided to the University of Sydney Laboratory of Veterinary Parasitology. A total of 27 frozen individual scat samples were received and stored at −20 °C until processing.

An additional four brown rats (*R. norvegicus*) caught and killed by a pet cat in Sydney’s Inner West (Summer Hill and Annandale) were donated for this study. It should be noted that these rat carcasses were in poor condition, with freezer burn and some organs missing. A necropsy was performed to retrieve any adult *Angiostrongylus* worms by teasing open the lung tissue and pulmonary arteries. The adult *Angiostrongylus* sp. was determined *via* light microscopy of the male bursa and spicules, and female terminal papilla (Bhaibulaya, 1968; Valentyne et al., 2020). An aliquot of faeces was collected from the colon of each rat for DNA isolation.

### 2.2 Molecular diagnostics and *Angiostrongylus* spp. and haplotype determination

Total genomic DNA from the scat samples (∼100 mg each) was isolated using the ISOLATE II Fecal DNA Kit (Bioline, Sydney, Australia) as per the manufacturerʼs instructions and eluted to a final volume of 80 μl. First, the hypersensitive AcanR3990 qPCR assay developed by Sears et al. (2020) was run using the same reagent preparation and cycling conditions described in Baláž et al. (2023) to determine the infection status of each rat/scat sample. *Angiostrongylus*-positive scat samples (AcanR3990 qPCR Ct ≤ 35) were then subjected to qPCR assay targeting a 250 bp *cox*1 region to produce amplicons for Sanger sequencing and subsequent species and haplotype determination (“Assay 2”; Mallaiyaraj Mahalingam et al., 2021). *cox*1 PCR products with sufficient amplification (Ct ≤ 35) and melt-curves corresponding to positive controls were sent to Macrogen (Seoul, South Korea) for purification and unidirectional Sanger sequencing using the forward primer (AngiCOI_forward [S0963]). All qPCR reactions were performed alongside positive (*A. cantonensis* L1), extraction and no-template controls.

The *cox*1 qPCR and sequencing were repeated on adult worm *A. cantonensis* tissue taken from the mid-section of a male specimen found in one of the Inner West rats (NC002). DNA was isolated from the tissue using the Monarch Genomic DNA Purification Kit (New England Biolabs, Melbourne, Australia) and eluted to 80 μl.

On the partial *cox*1 region amplified, there are three single nucleotide polymorphisms (SNPs) that distinguish the two locally known *A. cantonensis* haplotypes, Ac13 and SYD.1 (Mallaiyaraj Mahalingam et al., 2021). This region also contains SNPs, which allow for the distinction of *Angiostrongylus* at the species level. Sequences were manually trimmed for quality prior to alignment with published *cox*1 sequences of *A. cantonensis* Ac13 (Rodpai et al., 2016), *A. cantonensis* SYD.1 (Červená et al., 2019) and *A. mackerrasae* (Valentyne et al., 2020) *cox*1 sequences (KU532146.1, MK570631.1 and NC_046586.1, respectively) in CLC Main Workbench 22 (Qiagen).

### 2.3 Statistical analyses

The prevalence of *Angiostrongylus-*positive rats according to AcanR3990 qPCR was determined for the City of Sydney rats, the Inner West rats, and both groups of rats combined. Proportions with a 95% confidence interval (Wilson method) were calculated using the online calculator by Epitools (https://epitools.ausvet.com.au/ciproportion).

Due to missing morphometric data from the opportunistically collected Inner West rats (*n* = 4), only the city of Sydney rats (*n* = 27) were included in the following analysis carried out in R v 4.3 (R Core Team, 2023). To calculate a ‘Health Index’ (function of the rat’s weight according to size) of rats while controlling for rat species differences and sexual dimorphism, a General Linear Model (GLM), using the function “*glm*” from the package *MASS* (Venables and Ripley, 2002) was constructed. Weight was the response variable, species and sex were set as grouping factors, and tibia length was included as a covariate. This initial model included individual factors and 2-way and 3-way interactions. A Shapiro-Wilk test of normality, using the function “shapiro.test” from the package *stats* (R Core Team, 2023); Levene’s test for homogeneity of variance (homoscedasticity) using the function “leveneTest” from the package *car* (Fox and Weisberg, 2019), and Pearsonʼs dispersion test were used as diagnostics of the initial model. A log-transformation was applied to ‘weight’ due to non-normality and overdispersion of residuals. For model refinement, we used the function stepAIC from the package *MASS* (Venables and Ripley, 2002). We calculated the difference in corrected Akaike information criterion (AICc; Δm) between models and excluded models with Δm > 2 as having substantially less support (Burnham and Anderson, 2002). The final model included only tibia length. The residuals from the final model were termed as a ‘Health Index’ for each rat. Then, to estimate the association of rat species, sex, age and health index with infection status (i.e. positive or negative), a multinomial model was constructed using the function “multinom” from the package *nnet* (Venables and Ripley, 2002). The initial model included species, sex, health index and tibia length (as a proxy for age), their 2-way interactions, and the 3-way interactions between species, health index and tibia length and species, sex and tibia length. As before, for model refinement, we used the function stepAIC from the package *MASS* (Venables and Ripley, 2002). We calculated Δm between models and excluded models with Δm > 2 as having substantially less support (Burnham and Anderson, 2002). The final model included species, sex, tibia length, species by sex, species by tibia length and species by sex by tibia length. *P*-values were generated by the type III Wald chi-square test using the function “Anova” from the package *car* (Fox and Weisberg, 2019). Statistical significance was defined by α = 0.05.

## 3 Results

### 3.1 One of four *R. norvegicus* had adult worms present at necropsy

Of the four *R. norvegicus* opportunistically collected, two were male, and two were female. Upon necropsy, one rat (NC002) was positive for *Angiostrongylus* adults. Two worms, one male and one female, which matched morphological descriptions of *A. cantonensis*, were found.

### 3.2 Moderate prevalence of *A. cantonensis* haplotype SYD.1 in faeces of trapped rats

The city of Sydney scat samples (*n* = 27) originated from 21 *R. norvegicus* (15 male, 6 female) and six *R. rattus* (5 male, 1 female). Six of the 27 scat samples screened (22.2%; 95% CI: 10.6–40.8%) returned a positive result for *Angiostrongylus* according to AcanR3990 qPCR (Ct < 35; Table 1). Of these, four (AID003, AID005, AID008, AID009) could be identified as *A. cantonensis* haplotype SYD.1*,* according to partial *cox*1 sequences (Table 1).

**Table 1:** Summary of diagnostic results for the *Angiostrongylus*-positive rats (*Rattus norvegicus* and *Rattus rattus*) included in this study from Sydney, Australia.

ID	Location	Latitude, longitude	Species	Sex	Weight (g)	Tibia length (mm)	AcanR3990 Ct-value	*cox*1 Ct-value	*cox*1 species	*cox*1 haplotype
AID003	Hyde Park Sandringham Memorial	−33.872845, 151.211910	*R.norvegicus*	M	234	45.0	20.40	31.75	*A.cantonensis*	SYD.1
AID005	Royal Botanic Garden 3	−33.864526, 151.216392	*R.rattus*	M	201	47.2	20.06	31.42	*A. cantonensis*	SYD.1
AID007	Royal Botanic Garden 4	−33.864383, 151.216984	*R. rattus*	M	102	38.7	15.52	neg		
AID008	Eveleigh Garden St	−33.895644, 151.197249	*R. norvegicus*	F	298	50.9	18.71	28.74	*A. cantonensis*	SYD.1
AID009	Eveleigh Garden St	−33.895644, 151.197249	*R. rattus*	F	175	43.9	15.18	26.01	*A. cantonensis*	SYD.1
AID015	Eveleigh Green	−33.897141, 151.195129	*R. norvegicus*	M	322	52.4	34.88	37.44		
NC001	Annandale	−33.881454, 151.170188	*R. norvegicus*	M	197.4	NA	34.58	38.80		
NC002^a^	Annandale	−33.881454, 151.170188	*R. norvegicus*	F	171.9	NA	10.51	21.62	*A. cantonensis*	SYD.1
NC003	Annandale	−33.881454, 151.170188	*R. norvegicus*	F	81.8	NA	32.33	neg		

*Notes*: *Angiostrongylus* infection status was determined *via* faecal AcanR3990qPCR. Rats were either trapped in the city of Sydney (AID#) or opportunistically collected from the inner west (NC#). *Abbreviations*: Ct, cycle threshold; F, female; M, male; NA, not available.

a *cox*1 sequencing of this faecal sample was unsuccessful, but adult *Angiostrongylus* discovered during necropsy was available for molecular haplotype determination instead.

Additionally, three of the four Inner West *R. norvegicus* were positive (75%; 95% CI: 30.1–95.4%) upon AcanR3990 qPCR. None of these four faecal *cox*1 PCR products could be sequenced; however, partial *cox*1 amplification and subsequent sequencing of adult male worm DNA from the single rat positive at necropsy (NC002) revealed that it was harbouring the SYD.1 haplotype (Table 1).

Considering the infection status of all rats, including from the city of Sydney and the Inner West, the combined prevalence is 9/31, or 29% (95% CI: 16.1–46.6%).

### 3.3 *Angiostrongylus*-infected rats came from parks and residential suburbs

**Fig. 1:**
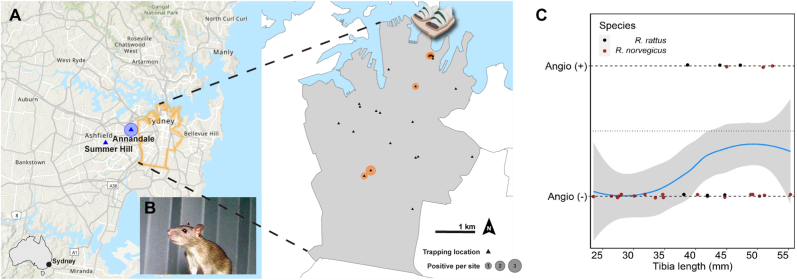
Location and *Angiostrongylus*-infection status of urban rats trapped in Sydney, Australia, and probability of *Angiostrongylus*-infection depending on rat tibia length (as a proxy for age). **A** Collection/trapping locations (triangles) and number of *Angiostrongylus*-positive rats per location (circles; various sizes) in the present study. Rats were trapped throughout the City of Sydney (*n* = 27; indicated in orange; magnified on the RHS) between 2020 and 2021. Several rats (*n* = 4) opportunistically collected during 2020–2023 Sydney’s Inner West (indicated in blue) were also included in the study. Infection status was determined using faecal AcanR3990 qPCR (Sears et al., 2020); samples with Ct ≤ 35 were considered positive. Left-most map provided by the City of Sydney https://www.cityofsydney.nsw.gov.au/areas-of-service. **B** Photo of a brown rat (*Rattus norvegicus*) captured on camera in a residential property in the Inner West of Sydney. Credit: Dr Anne Quain. **C** Probability (blue line ± SEM) of a rat from the City of Sydney being *Angiostrongylus*-positive (1) or negative (0) according to their age, using tibia length (mm) as a proxy. Each dot represents an individual rat, with black dots being black rats (*R. rattus*) and brown dots being brown rats (*R. norvegicus*). The horizontal dotted line represents a probability of 0.5, while the two dashed lines represent probabilities of 1 (Angio +) or 0 (Angio –).

All *Angiostrongylus*-positive rats from the city of Sydney were trapped at one of the following three public parks: Royal Botanical Gardens (*n* = 2), Hyde Park (*n* = 1) and Eveleigh Green/Garden St (*n* = 3) (Table 1; Fig. 1A). The three *Angiostrongylus*-positive rats from the Inner West were collected in Annandale, a leafy residential suburb directly west of the city of Sydney (Table 1; Fig. 1A and B).

### 3.4 Older rats are more likely to be *Angiostrongylus*-positive

Only tibia length (proxy of age) was associated with infection status (*χ*^2^_1_ = 5.331, *P* = 0.021). There was no association between infection status and species (*χ*^2^_1_ = 0.070, *P* = 0.792), sex (*χ*^2^_1_ = 2.050, *P* = 0.152), species by sex (*χ*^2^_1_ = 0.022, *P* = 0.882), species by tibia length (*χ*^2^_1_ = 0.139, *P* = 0.709), or species by sex by tibia length (*χ*^2^_1_ = 2.336, *P* = 0.311). Rats with longer tibia length, i.e. older, were significantly more likely to have a positive infectious status than younger rats (Fig. 1C).

## 4 Discussion

This survey unequivocally demonstrates that invasive rats (both *R. rattus* and *R. norvegicus*) in Sydney commonly act as natural hosts for *A. cantonensis*. Although alarming, this is unsurprising as rats elsewhere along eastern NSW and Queensland (QLD) have reportedly been infected (Table 2). Undoubtedly, the co-occurrence of patently infected rats with susceptible molluscan intermediate hosts creates a scenario conducive to accidental definitive host infection, which has been observed in humans and domestic dogs in Sydney previously (Senanayake et al., 2003; Lunn et al., 2012; Morton et al., 2013; Berkhout et al., 2019; Lee et al., 2021; Mallaiyaraj Mahalingam et al., 2021). Infected rats being trapped in public parks, gardens and residences in this study is concerning; the residents of Sydney, such as dogs being walked, curious toddlers, or people eating from home vegetable gardens are at risk if susceptible molluscs are present. Impressively, *A. cantonensis* can be found in rats across various continents and habitats, oftentimes where humans are densely populated - which raises concerns for human, companion animal, and wildlife health. Very high prevalence levels have been recorded in pestiferous rats in Hawaii (93.9%; Jarvi et al., 2017) and Rio de Janeiro, Brazil (71%; Simões et al., 2014). Moderate *A. cantonensis* prevalence levels have been reported in the Ogasawara archipelago, Japan (33.8%), Atlanta, USA (21.2%), and Valencia, Spain (20%) (Tokiwa et al., 2013; Galán-Puchades et al., 2023; Gottdenker et al., 2023). The detected prevalence in Sydney (29%), according to our study, appears to be comparable to these regions with historical introduction of *A. cantonensis*.

**Table 2:** Prevalence of *Angiostrongylus* spp. reported in *Rattus* spp. in Australia.

State	Location	Prevalence % (Positive/No. examined)	Reference
Queensland (QLD)	Brisbane City	20.68% (67/324)	Bhaibulaya (1968)
	Rathdowney	0% (0/37)	
	Mt. Spec	0% (0/80)	
	Mt. Glorious	8.53% (11/129)	
	Cairns	0% (0/27)	Aghazadeh et al. (2015b)
	Brisbane	18.13% (68/375)	
	Brisbane	4.88% (8/164)	Mackerras and Sandars (1955)
	Innisfail	11.76% (2/17)	
	Mt. Nebo	0% (0/2)	
	Benarkin	0% (0/18)	
New South Wales (NSW)	North Jervis Bay	4.11% (33/802)	Stokes et al. (2007)*
	South Jervis Bay	7.91% (17/215)	
	Sydney	29.03% (9/31)	Present study (2024)**
	Western Sydney	64.29% (9/14)	Rivory et al. (2023)**

*Notes*: The method of *Angiostrongylus* detection for all studies included necropsy; references indicated with one asterisk (*) included faecal Baermann test, and those indicated with two asterisks (**) included molecular methods.

Our previous work at a zoo in western Sydney (approximately 30 km west of the city of Sydney) demonstrated that 9/14 (64%) of trapped *R. norvegicus* were infected with *A. cantonensis* (Rivory et al., 2023). The prevalence in the present study is considerably lower, highlighting the random variation in *A. cantonensis* abundance between complex host-parasite systems from one habitat to another (Niebuhr et al., 2021; Rollins et al., 2021).

The significance of tibia length as a predictor in our model supports the concept that older rats are more likely to have been exposed to the parasite at some point during their lifespan, possibly even multiple times (Simões et al., 2014; Niebuhr et al., 2021). This contrasts with Jarvi et al. (2017), who reported a negative correlation between weight (another proxy for age) and adult worm burden in Hawaiian *R. rattus*. Their finding aligns with the hypothesis that rats can regulate parasite burdens upon re-infection (Au and Ko, 1979). However, our study focused solely on infection status, not worm burden. Additionally, Sydneyʼs rat population had just experienced a crash (Bedoya-Pérez et al., 2021), likely resulting in a younger population encountering the parasite for the first time rather than being re-infected.

Most surveys of Australian rats demonstrate that *R. norvegicus* is infected with *Angiostrongylus* spp. at a higher prevalence than *R. rattus* (Mackerras and Sandars, 1955; Bhaibulaya, 1968; Aghazadeh et al., 2015b). Although rat species was not significant in our model, a higher proportion of *R. rattus* were infected. Despite both species of rat being generalist omnivores, perhaps (i) *R. rattus* preference for wild vegetation (Cox et al., 2000; Adams et al., 2023) and (ii) aggressive competition from highly urban *R. norvegicus* (King et al., 2011) would drive *R. rattus* in Sydney to denser natural habitats (such as parks and gardens) where gastropods as a food source are abundant.

Considering the poor condition of the four Inner West *R. norvegicus* carcasses necropsied in this study, the failure to find adult *Angiostrongylus* in 2/3 faecal AcanR3990-positive individuals may have been due to post-mortem artefacts. Alternatively, these necropsy-negative rats may have had a positive faecal AcanR3990 detection due to the passage of pre-patent larvae.

The five individual rats with successful partial *cox*1 amplification and sequencing (faecal DNA, *n* = 4; adult worm DNA, *n* = 1) were all found to be the *A. cantonensis* SYD.1 haplotype. In our previous work in western Sydney, most trapped *R. norvegicus* and opportunistically collected rat faeces contained the Ac13 haplotype, with some rats co-infected with both haplotypes (Rivory et al., 2023). The reliance on Sanger sequencing and the small number of clean sequences available for analysis in the present study may contribute to this finding. The differences between the endemic SYD.1 and Ac13 haplotypes regarding their pathogenicity, host preferences and epidemiology have not yet been determined, so the reason for this observation is uncertain. Limited dispersal due to habitat fragmentation in rodent and gastropod communities might restrict the spread of introduced haplotypes (Bordes et al., 2015), therefore preventing co-occurrence of Ac13 and SYD.1 in some areas.

Similarly, our analysis of partial *cox*1 sequences did not detect any presence of *A. mackerrasae*. This finding suggests a possible absence of *A. mackerrasae* in Sydney’s urban rat population. The small sample size and use of first-generation sequencing comes with its limitations; however, *A. mackerrasae* may have some degree of host specificity, as it has been found to infect native bush rats (*Rattus fuscipes*), swamp rats (*Rattus lutreolus*) and *R. norvegicus*, but not *R. rattus* (Bhaibulaya, 1968). Additionally, the only locations where *A. mackerrasae* has been found to date are in QLD (Bhaibulaya, 1968; Aghazadeh et al., 2015b), Tasmania (Spratt, 2015) and in coastal forests over 130 km south of Sydney (Stokes et al., 2007). While the distribution of *A. mackerrasae* so far seems to reflect the natural range of its preferred host, *R. fuscipes*, uncertainties remain (Aghazadeh et al., 2015a). Recently, effort was made to confirm the culprit species in several canine angiostrongyliasis cases using ITS-2 deep sequencing, with no cases detected thus far (Baláž et al., 2023). Detection of co-infection with more than one haplotype of *Angiostrongylus* in rat faeces and accidental host cerebrospinal fluid could be resolved by developing a *cox*1 next-generation sequencing protocol.

## 5 Conclusions

Overall, we confirmed that a substantial proportion of pestiferous black (*R. rattus*) and brown (*R. norvegicus*) rats in Sydney, Australia, are infected with *A. cantonensis.* Concerningly, infected rats were found around public parks and residential areas. Tibia length of rats was a significant predictor for *Angiostrongylus* infection, which indicates that older rats in this study were more likely to be infected. In this rat population, only *A. cantonensis* haplotype SYD.1 was found. While the specific implications of the dominant SYD.1 haplotype are not yet understood, we have established the parasiteʼs existence in the metropolitan city of Sydney. Given the publicʼs limited awareness of rat lungworm, increased educational efforts are crucial to help people take measures to protect themselves and their pets.

## Funding

This work was supported by the 10.13039/501100001774University of Sydney Research Training Program (RTP) Tuition Fee Offset, Sydney, NSW. Rat trapping was funded by the Council of the City of Sydney.

## Ethical approval

Animal ethics approval for rat sampling in a separate project was granted by the 10.13039/501100001774University of Sydney Animal Ethics Committee (approval number 2020/1725, N00/1–2013/3/5920, 2017/1130 and 2019/1665). Ethics approval was obtained by Bedoya-Pérez et al. (2023).

## CRediT authorship contribution statement

**Phoebe Rivory:** Conceptualization, Data curation, Formal analysis, Investigation, Methodology, Project administration, Writing – original draft, Writing – review & editing. **Miguel Bedoya-Pérez:** Resources, Data curation, Formal analysis, Writing – review & editing. **Michael P. Ward:** Data curation, Formal analysis, Writing – original draft, Writing – review & editing. **Jan Šlapeta:** Project administration, Resources, Supervision, Writing – review & editing.

## Declaration of competing interests

The authors declare that they have no known competing financial interests or personal relationships that could have influenced the outcomes of this study.

## Data Availability

All data, including a master table, statistical analyses and partial *cox*1 sequence alignment, is available online at LabArchives (https://dx.doi.org/10.25833/7ppn-gs90).
